# Exploring the Potential Use of Virtual Reality with a Supraorbital Keyhole Craniotomy for Anterior Skull Base Meningiomas: Two Case Reports

**DOI:** 10.3390/jpm14111074

**Published:** 2024-10-25

**Authors:** Jose Valerio, Maria P. Fernandez Gomez, Arturo Ayala Arcipreste, Noe Santiago Rea, Penelope Mantilla, Immanuel O. Olarinde, Andres M. Alvarez-Pinzon

**Affiliations:** 1 Department of Neurosurgery Oncology and Radiosurgery, Miami Neuroscience Center, Larkin Community Hospital, Miami, FL 33143, USA; jevalerio@jvaleriomd.com; 2 Department of Neurological Surgery, Palmetto General Hospital, Hialeah, FL 33016, USA; 3 Department of Neurological Surgery, Latinoamerica Valerio Foundation, Weston, FL 33331, USA; mariapaulafg96@gmail.com (M.P.F.G.); neurocx.online@gmail.com (A.A.A.); penelopemantilla@gmail.com (P.M.); olarindei@yahoo.com (I.O.O.); 4 Division of Research, Institute for Human Health and Disease Intervention, FAU Charles E. Schmidt College of Medicine, Florida Atlantic University, Boca Raton, FL 33431, USA

**Keywords:** anterior cranial fossa, case series, craniotomy, eyebrow, meningioma, minimally invasive surgical procedures, skull base

## Abstract

Introduction: A supraorbital keyhole craniotomy (SOKC) is a novel alternative to frontal craniotomies for accessing the anterior fossa for resecting tumors and clipping aneurysms; however, its implementation is limited in patients at a high risk of complications. We present two cases involving the use of augmented reality (AR) and virtual reality (VR) for patient selection and preoperative planning for a supraorbital tumor resection of anterior fossa meningiomas. Methods: This is a prospective, single-center case series at a research institute. We identified patients with an anterior or middle fossa meningioma regardless of age, gender, and tumor characteristics who could undergo an SOKC and MRI. The preoperative planning was performed with the BrainLab Magic Leap AR/VR platform. The meningiomas were resected through the SOKC under neuronavigation. Results: We identified two cases: a 37-year-old male with a meningioma in the sellar region and an 84-year-old male with a right anterior fossa meningioma, both confirmed by MRI. Both patients had a complete tumor resection by a minimally invasive SOKC after preoperative planning with the AR/VR platform. Postoperatively, hyponatremia complicated the first case, while the second case developed an intracranial hemorrhage. They both recovered after the appropriate interventions. Conclusions: The use of an SOKC for anterior skull base meningiomas should be individualized after considering the lesion characteristics, vascular control needs, and the surgeon’s expertise. VR/AR-assisted preoperative evaluation and planning will optimize the patient selection and surgical outcomes. We can utilize VR/AR technologies to identify patients that will benefit from an SOKC and expand the implementation of the approach beyond its current limitations.

## 1. Introduction

Meningiomas are the most common primary brain tumors, accounting for almost 38% of all central nervous system tumors [1,2]. They have an estimated incidence rate of 8.3 cases per 100,000 individuals [2,3]. Surgical resection is the standard treatment for meningiomas, particularly in cases of progressive disease, neurological deficits, or significant vasogenic edema. However, a traditional craniotomy’s invasive nature presents considerable limitations in surgical planning and execution, especially for elderly patients and those with multiple comorbidities [4]. The excessive tissue exposure and retraction associated with traditional craniotomies increase postoperative morbidity, highlighting the need for less invasive approaches [5].

One such approach is the supraorbital keyhole craniotomy (SOKC) introduced by Perneczky and his colleagues [5,6]. A small “keyhole” skin incision and craniotomy is made through the eyebrow or palpebral fissure to access the anterior cranial fossa [5]. With proper patient positioning and relaxation, a piecemeal total resection with minimal brain retraction and manipulation is often achieved [5]. Thakur and colleagues demonstrated that keyhole surgeries achieved meningioma resection rates comparable to traditional craniotomies without increasing complication rates [7].

Despite several advantages, an SOKC has its limitations. The small size of the craniotomy restricts surgical field exposure and increases blind spots [5,8,9]. In addition, the technicalities associated with a confined range of movement may protract the learning curve compared to traditional craniotomies [5]. Therefore, an emphasis on the optimal patient selection, comprehensive preoperative planning, and ideal intraoperative navigation is gaining traction in recent years. Advances in computer tomography (CT) and magnetic resonance imaging (MRI) have improved the visualization of the critical neuroanatomical structures, albeit in poorly maneuverable 2D representations. The advent of microsurgical instruments and high-definition endoscopes improves the visualization and lighting to overcome the restrictions of an SOKC [5,8,9], but the surgeon must look at a screen away from the operating field and transfer three- and four-dimensional (3/4D) virtual anatomic details—obtained from preoperative scans—to the operating field. However, through augmented reality (AR), virtual images are overlayed on the real operating field to improve the efficiency of the neuronavigation [10].

Consequently, there is a critical need to explore and develop better minimally invasive surgical strategies utilizing innovative technologies like 3/4D AR and virtual reality (VR) for preoperative planning, which could enhance success rates and reduce both the complication rates and duration of surgery to improve the outcomes for patients with various comorbidities [11].

The SOKC is becoming a valuable alternative approach with a notable reduction in patient morbidity and hospital stays, while achieving comparable tumor resection rates [12]. This minimally invasive technique allows strategic access to the brain through a small eyebrow incision [13]. An SOKC facilitates direct and precise access to the frontal and temporal lobes with minimal brain retraction and less damage to the adjacent healthy tissue [6,14,15]. Advances in VR and AR technologies have introduced a wide range of applications for improved visualization and navigation in neurosurgery [16]. Mixed Reality (MR), an amalgamation of VR and AR, holds promise for enhancing minimally invasive neurosurgical techniques by giving surgeons a comprehensive view of virtual and real elements during surgical procedures [17].

In this paper, we present two cases involving the use of MR in the preoperative planning of the minimally invasive resection of anterior skull base meningiomas through an SOKC to illustrate the role of MR in improving the efficacy and safety of a novel surgical approach. Furthermore, we provide a detailed description of the surgical technique, shedding light on its potential for optimizing surgical outcomes and enhancing patient care.

## 2. Methods

### 2.1. Study Design and Participants

This is a prospective, single-center study with two case reports conducted at a neuroscience research institute. We included patients diagnosed with meningioma located in the anterior or middle cranial fossa regardless of age, gender, and other tumor characteristics. We then excluded patients with previous cranial surgeries, contraindications to an SOKC (such as an unfavorable tumor location, significant frontal lobe edema, and coagulopathy), or an inability to undergo MRI. We collected demographic details; medical history; tumor characteristics; preoperative symptoms; preoperative imaging; surgical details, including the application of 3D and 4D technologies; and the immediate postoperative outcomes.

### 2.2. Presurgical Planning

**Figure 1:**
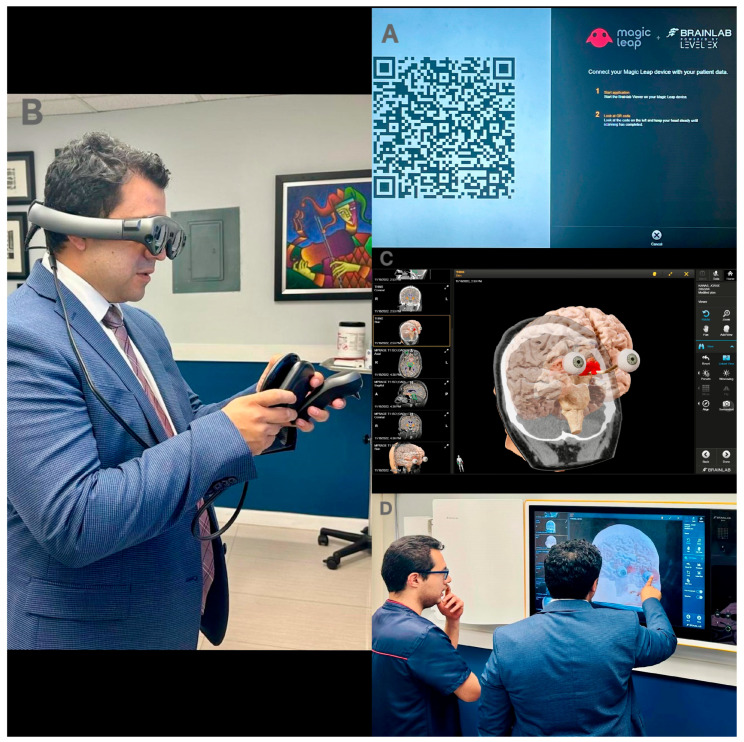
Preoperative planning with VR. (**A**) BrainLab QR code. (**B**) The Magic leap device is used to scan the QR code. (**C**) Patient data are linked to the platform to generate 3D images. (**D**) The 3D images are used for preoperative surgical planning.

Figure 1 shows the comprehensive planning with the 3D images reconstructed by the BrainLab (manufactured by BrainLab AG in Munich, Germany) Magic Leap 1 (manufactured by Magic Leap Inc., Plantation, FL, USA) VR/AR platform, (Figure 1A–D). We used the 3D images to plan the ideal surgical approach by analyzing the tumors’ relationships to surrounding structures. For the anterior skull base tumors, we suggested considering the relationship with the frontal sinus to avoid injury during the craniotomy. Moreover, it was important to identify the medial and lateral landmarks of the craniotomy sufficient for proximal and distal vascular control. We suggested proceeding with the SOKC for anterior and middle skull base lesions proximal to or within the sellar region, as those located more posteriorly pose challenges for vascular control and are too deeply located for this approach.

### 2.3. Neurosurgical Intervention

Table 1 shows the detailed surgical procedure description.

With the patient supine on the operating table under general anesthesia, the head was fixed in a three-pin Mayfield. The single pin of the head fixator was placed in the opposite frontal area to allow free manipulation on the ipsilateral side during the procedure. The neck was extended to position the head above the heart to facilitate venous drainage during surgery. This positioning provides optimal access to the anterior skull base and minimizes the risk of retraction-related injuries to the brain and neurovasculature.

A skin incision was made through the unshaved eyebrow for superior aesthetic results while providing adequate exposure for tumor resection. Then, we performed a standard subcutaneous dissection with monopolar electrocautery, followed by a subperiosteal dissection to detach the galea from the underlying squamous portion of the frontal bone. The incision was centered over the lateral eyebrow and followed the eyebrow hair direction, extending 4–5 cm in length. This precise dissection technique minimizes tissue trauma and facilitates the optimal visualization of the underlying neurovascular structures, minimizing the risk of frontal numbness.

We made a D-shaped craniotomy (with the linear aspect positioned downward along the floor of the anterior cranial fossa) measuring 2.5 cm × 2 cm (the dimensions may vary depending on the tumor size and location) for optimal access to the target area with minimal risk of injury to critical neurovascular structures. After the craniotomy, we meticulously drilled the inner surface of the orbital portion of the frontal bone to widen the operating field for opening the dura and subsequently resecting the tumor. The dura mater was opened in an oblique or cross fashion for the direct visualization of the tumor borders and boundaries. After gently retracting the dura, the tumor was exposed using Rhoton forceps, cotton balls, and cotonoids to preserve the delicate neurovascular structures, including the olfactory nerve, optic nerve, and critical vessels. We applied microsurgical principles to minimize tissue trauma and optimize the surgical field. The arachnoid layer was opened to decrease intracranial pressure and further enhance the surgical field.

We dissected the tumor with the aid of neuronavigation, which provided real-time imaging guidance to accurately navigate the complex neuroanatomy. The tumor was dissected in a plane between the capsule and the surrounding normal tissue, preserving as much of the arachnoid and pia mater as possible. We used delicate microsurgical techniques to completely remove the tumor with minimal damage to the neurovascular structures. After resecting the tumor, we achieved hemostasis with bipolar cautery, absorbable hemostatic agents, and bleeding vessel suture ligation. We approximated the dura mater and reconstructed the bone flap with titanium plates and screws. We closed in layers to minimize the risk of CSF leakage and postoperative complications.

### 2.4. Consent and Waiver

The participants and any identifiable individuals consented to the publication of his/her image. Consent was obtained or waived by all participants in this study. The Larkin Healthcare System issued approval LCH-8-062021.

This case series has been reported in line with the PROCESS Guidelines [18].

## 3. Results

We identified two patients with anterior or middle skull base meningioma amenable to an SOKC. Both patients had no previous cranial surgeries, and no contraindications to an SOKC and MRI.

### 3.1. Case 1

#### 3.1.1. Demography, Presentation, and Evaluation

**Figure 2:**
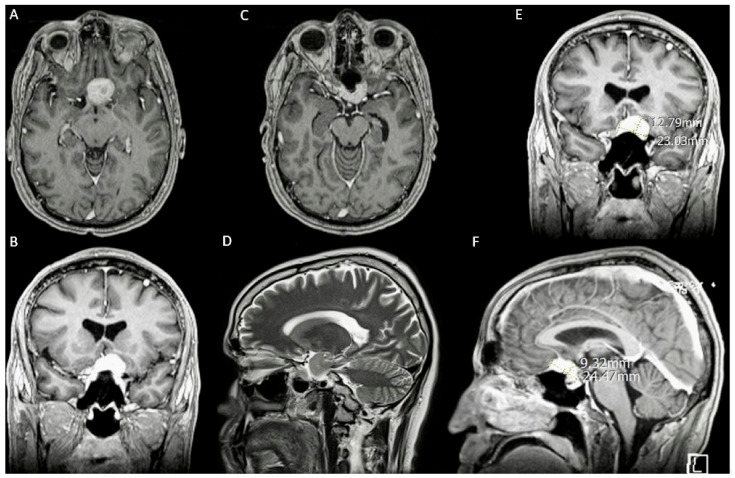
Case 1’s preoperative MRI. (**A**) An axial T1 view of a 1.5 cm × 2.1 cm enhancing sellar lesion. (**B**) A coronal T1 view of the suprasellar mass encircling the optic chiasm. (**C**) An axial T1 view of the mass compressing the optic chiasm and left carotid artery and encircling the left optic nerve. (**D**) A sagittal T2 sequence showing the close relationship of the tumor with the terminal branches of the ICA. (**E**) Coronal T1 view of tumor dimensions. (**F**) Sagittal T1 view of tumor dimensions.

A 37-year-old male with a 6-month history of progressively worsening blurry vision in the left eye. Physical examination revealed a significant peripheral visual field defect. A brain MRI with and without contrast (Figure 2) revealed an enhancing sellar lesion compressing the left optic nerve, internal carotid artery, anterior cerebral artery, and optic chiasm (Figure 2A–F).

#### 3.1.2. Intervention

**Figure 3:**
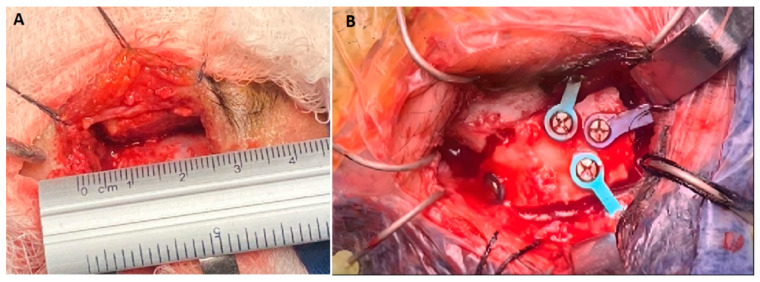
Intraoperative images of supraorbital approach. (**A**) Minimally invasive supraorbital keyhole craniotomy measuring 2.5 × 2.0 cm. (**B**) Cranioplasty after tumor resection.

We decided that an SOKC through a sub-frontal approach was suitable based on the suprasellar location and lateral extension of the tumor, the higher risk of cerebrospinal fluid leakage associated with an endonasal approach, and the surgeon’s preference. In addition, the relationship between the supraorbital ridge and the frontal sinus and the distance between the tumor and the inner table of the skull seen on the BrainLab Magic Leap platform favored this approach. With neuronavigation guidance, we resected the tumor through a minimally invasive supraciliary craniotomy measuring 2.5 × 2.0 cm (Figure 3A,B).

#### 3.1.3. Postoperative Period

**Figure 4:**
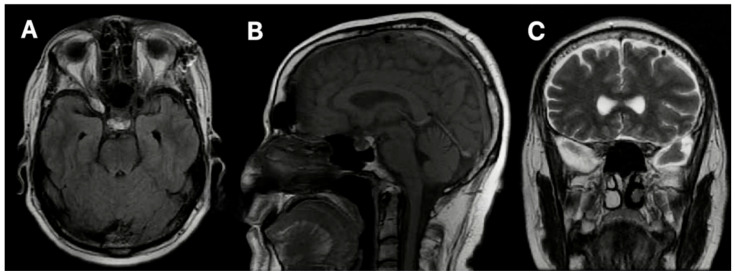
Case 1’s postoperative MRI shows no residual tumor but mild frontal pneumocephalus, which is better appreciated on the sagittal view. (**A**) Axial view. (**B**) Sagittal view. (**C**) Coronal view.

A postoperative MRI (Figure 4) showed the complete tumor resection. However, we observed mild frontal pneumocephalus, a common occurrence following minimally invasive cranial procedures, which typically resolves spontaneously (Figure 4A–C). There was no radiographic evidence of an intracranial hemorrhage.

Five days postoperatively, the patient developed hyponatremia, which manifested as lower extremity muscle pain. We ruled out renal salt wasting and the syndrome of inappropriate antidiuretic hormone secretion (SIADH) with appropriate laboratory studies. The patient’s serum sodium was corrected, and the symptoms resolved. The rest of the recovery was smooth. Vision was restored to his left eye, and there were no postoperative neurologic complications and CSF leaks.

### 3.2. Case 2

#### 3.2.1. Demography, Presentation, and Evaluation

**Figure 5:**
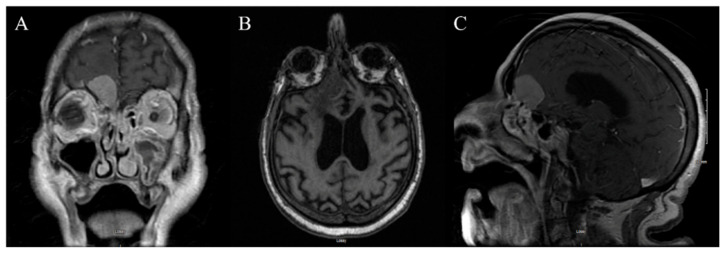
Case 2’s preoperative contrast-enhanced MRI of a dura-attached enhanced 3.2 × 2.4 cm lesion in the anterior cranial fossa suggestive of a meningioma. (**A**) Coronal view shows compression of the basal surface of the frontal lobe. (**B**) Axial view shows blunted anterior horn of the right lateral ventricle. (**C**) Sagittal view shows compression of the basal surface of the frontal lobe.

An 84-year-old male nursing home resident with a known history of dementia presented with persistent headaches. An initial evaluation showed left-sided weakness, which prompted a head CT scan. The CT scan showed hypo-attenuated white matter and absent grey–white matter differentiation in the left frontal lobe. A subsequent brain MRI (Figure 5) revealed an enhancing mass in the floor of the right anterior fossa, highly suggestive of a meningioma (Figure 5A–C).

#### 3.2.2. Intervention

We decided to proceed with a stereotactic right SOKC resection using neuronavigation given the tumor’s superficially anterior location. The preoperative planning with the BrainLeap Magic Lab platform highlighted the tumor’s proximity to the olfactory groove and the terminal branches of the anterior cerebral artery (ACA). The procedure was successfully performed without any intraoperative complications.

#### 3.2.3. Postoperative Period

**Figure 6:**
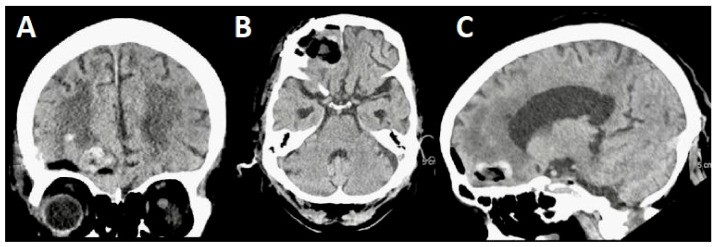
Case 2’s postoperative day 1 MRI showing a small amount of blood in the resection cavity with minimal compression of the frontal lobe. (**A**) Coronal view. (**B**) Axial view. (**C**) Sagittal view.

On the first postoperative day, the patient became unresponsive to verbal stimuli and only withdrew to pain. An urgent CT scan (Figure 6) revealed a small amount of blood within the cavity left after tumor resection and mild compression of the frontal lobe (Figure 6A–C).

We immediately started hyperosmotic fluid, oxygen therapy, hyperventilation, and other supportive care to decrease the intracranial pressure and optimize the cerebral perfusion. On postoperative day 18, the patient was discharged to a rehab facility without new neurologic deficits but required assistance due to his baseline cognitive impairment.

## 4. Discussion

### 4.1. Uses of VR and AR in Minimally Invasive Surgery

**Figure 7:**
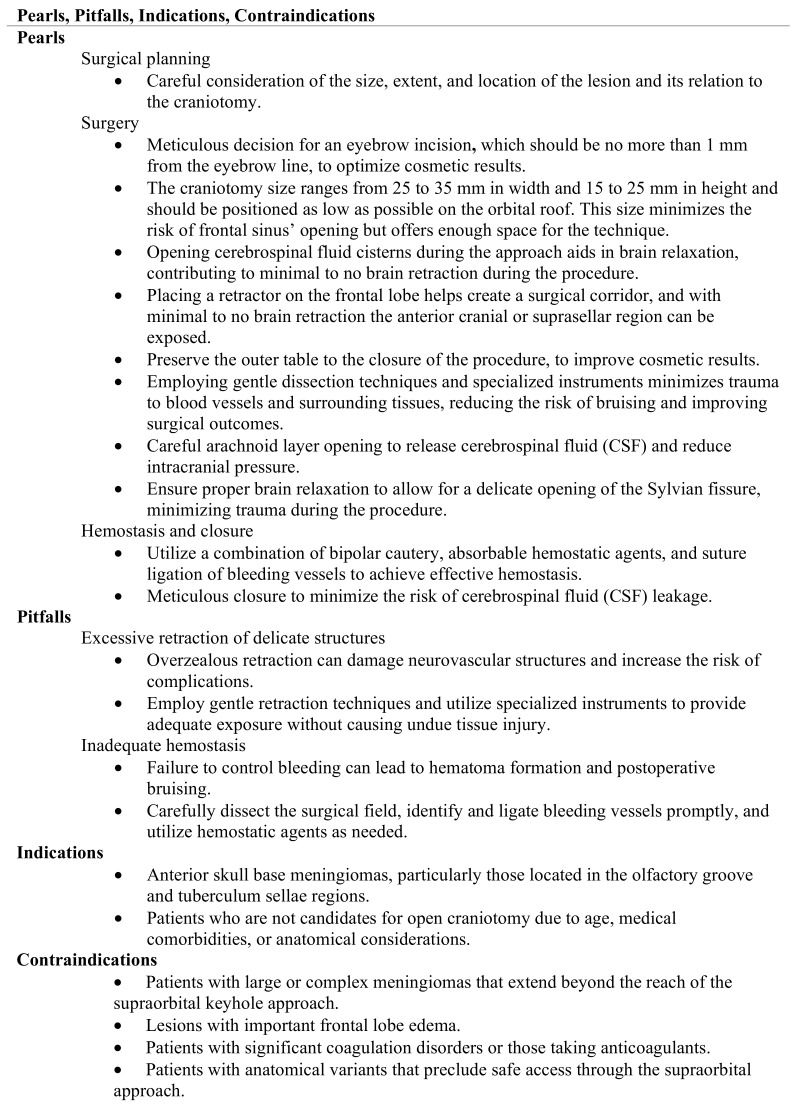
Pearls, pitfalls, indications, and contraindications of supraorbital craniotomy for meningioma resection.

In neurosurgery, AR is a valuable tool to enhance the identification of microsurgical anatomy [19]. In minimally invasive surgeries (MISs), the implementation of AR technology has shown potential benefits, enabling surgeons to avoid unnecessary manipulation and reduce the risk of injury to vital structures within confined surgical fields [10,20]. During surgical pre-planning involving 3D technology, different data are collected from sets of images with both anatomic and physiological information; rendering and modeling are then used to build part of a virtual body, so it can be studied and manipulated in different angles before surgery [21]. The 3D reconstruction may help during decision-making, as the preoperative planning may reduce surgical time and increase accuracy [22]. We believe that, in our cases, the planning with VR technology enabled us to identify the SOKC as the most suitable approach after considering patient-specific pros, cons, indications, and contraindications (Figure 7).

### 4.2. Supraorbital Keyhole Craniotomy

In recent years, the SOKC has emerged as a prominent minimally invasive procedure with significant advantages in managing vascular and malignant lesions (Table 2).

In conjunction with an endoscope, this technique enables the successful resection of deeply seated brain lesions [25,26]. Numerous clinical studies have unequivocally demonstrated the comparable effectiveness of SOKCs to conventional craniotomies in achieving complete resection with concurrently low complication rates, which reduces the financial burden of postoperative complications but also facilitates shorter recovery periods for patients [14,27,28,29]. A recent meta-analysis, comprising 124 studies and 8241 surgical cases, reported an 83.6% technical success rate for SOKCs, indicating either complete tumor resection or complete aneurysm clipping [12]. Notably, vascular pathology presents the greatest technical challenges, due to the smaller surgical field, imposing greater difficulties in attaining proximal and distal vascular control [23,30].

The primary outcomes after tumor resection via the supraorbital approach are favorable, characterized by low surgical morbidity, minimal blood loss, shortened hospital stays, and accelerated recovery periods [28,31]. However, possible complications should not be overlooked [12,28,31]. Furthermore, to acquire ample surgical proficiency in this technique, judicious patient selection is essential for minimizing the incidence of complications. According to Wilson and Ormond, the most amenable tumors for SOKCs are tuberculum sellar and olfactory groove meningiomas [13,31]. Lemée et al. found that age is a predictive factor for an intracranial hemorrhage after a meningioma resection as seen in the second case, our 84-year-old patient [32]. However, the use of MR may have minimized the extent of the bleed and curtailed poor sequelae.

The SOKC has also proven effective in treating gliomas and metastases in the anterior cranial fossae, perisylvian regions, and parasellar regions [11,27]. Ottenhausen et al. developed an algorithm that assists in recommending either a supraorbital approach or traditional bicoronal or pterional craniotomies, recognizing the variability and subjectivity involved in selecting the ideal tumor for a supraciliary craniotomy [33]. Thus, it is hypothesized that, in the future, SOKCs will be applied to a broader range of pathologies because of ongoing advancements and a refined learning curve [14,34].

### 4.3. VR/AR-Assisted Supraorbital Keyhole Craniotomy

The decision to perform an SOKC depends on several factors, including the specific characteristics of the lesion, the patient’s medical history, comprehensive imaging studies, the need to establish proximal and distal vascular control, and the surgeon’s proficiency with this surgical technique. AR and VR potentially make this decision easier.

#### 4.3.1. Patient Selection

The list of the types of meningiomas amenable to resection through SOKCs has evolved over the years. Meningiomas in the olfactory groove, superficial sella, and clinoid above the lesser wing favor an SOKC [7]. Although case 1 had a tumor that could be resected endonasally, the lateral extension of the tumor prolonged that approach and increased the risk of damaging delicate structures. With the help of VR, we were able to accurately determine the short distance between the inner border of the frontal bone and the tumor, which also favored an SOKC. Although VR and AR may play limited roles in choosing an approach in clearcut situations as in case 2, they can supplement the information from 2D images when deciding the approach for ambiguous tumors as in case 1. Furthermore, MR-generated simulations of the surgery can offer insights on the likely success of our approach based on patient-specific characteristics and expand the tumor types that can be resected through SOKCs. The second case also emphasized the challenges associated with managing elderly patients with meningiomas and comorbidities, such as dementia. Incorporating AR and VR into surgical planning will ensure we only operate on patients where the benefits of surgery far outweigh the risks. In the presented cases, the successful tumor resection using preoperative VR/AR planning and a subsequent SOKC highlights the importance of an improved and tailored surgical planning.

#### 4.3.2. Intraoperative Considerations

The use of VR and AR during surgery may resolve some technical difficulties. For instance, placing an endoscope through the craniotomy to illuminate deeper parts of the surgical field can further narrow an already small opening making the maneuverability of the microsurgical instruments more difficult. However, placing virtual images on a real neuroanatomy with AR allows the surgeon to see through the cranium without an endoscope, thereby maximizing the efficiency of the craniotomy [10].

The recurrence rate of certain types of meningiomas, such as petroclival meningioma, is still high due to the low rate of GTR, because of their invasiveness and adherence to neurovascular structures. Even the use of an endoscope in transcranial keyhole surgeries facilitated only an additional 55% tumor removal, highlighting the limitations of the current neuronavigational systems [7]. By integrating promising microsurgical approaches with advanced and innovative imaging and navigation techniques, neurosurgeons can strive to achieve the maximal tumor resection while preserving critical neural and vascular structures, ultimately enhancing patient outcomes in the field of neurosurgical oncology.

#### 4.3.3. Surgical Learning Curve

The experience and preference of surgeons can influence the outcomes of surgical procedures. Studies have shown that the operating time associated with SOKCs is comparable to traditional craniotomies, although the duration of surgery can be prolonged in larger tumors likely due to increased time spent resecting the tumor [35]. It can be argued that the back-and-forth motion of the surgeon between the neuronavigation display and the surgical field might contribute to the prolonged duration of neurosurgeries. The mobile, handheld, or head-mounted displays associated with AR-assisted surgeries may cut down this extra time. In addition, simulating the surgery in MR can adequately prepare surgeons, boost confidence, and shorten the learning curve for MISs [10].

#### 4.3.4. Accessibility and Ethics

Despite the potential benefits of MR-assisted keyhole surgeries, global accessibility and cost-effectiveness is of utmost importance. As we are discovering how to seamlessly integrate VR and AR to improve patient outcomes after neurosurgery and expand the indications for MISs, we should also prioritize making these technologies available to low-resource areas at affordable prices [36]. The use of advanced technologies should not significantly increase the financial burdens of neurosurgical procedures on patients. A global effort tailored at maintaining the highest adherence with ethical principles must also be in place [37].

### 4.4. Limitations and Future Directions

The patients’ baseline and postoperative neurologic status were not objectively assessed with a standardized tool to minimize bias. The underlying cognitive impairment in the second case may be a confounder in assessing the true impact of the intracranial bleed he suffered. In addition, we did not report the long-term postoperative outcomes of the patients.

Even though several applications of MR in neurosurgery have been documented, the evidence for their use in clinical settings is weak [10]. Since much evidence already exists comparing MISs with traditional craniotomies, the focus should be on gathering evidence on the integration of various VR/AR technologies with current neuronavigational systems in MISs. We should design randomized clinical trials comparing VR/AR-assisted MISs to traditional MISs. These studies should be designed with long-term follow-up in mind to elucidate other benefits or downsides of VR/AR-assisted MISs. It will be interesting to also study the impact of VR/AR on traditional craniotomies to improve patient outcomes in settings where microsurgical instruments are unavailable. Because the data for large comparative retrospective studies, systematic reviews, and meta-analyses are lacking, physicians should be encouraged to comprehensively narrate their experiences integrating these technologies into their practice. Large observational studies comparing outcomes in AR/VR-assisted surgeries with those where AR/VR technologies were not employed are also warranted.

## 5. Conclusions

The SOKC, as a neurosurgical approach, has been progressively validated through empirical evidence to offer outcomes that align closely with, if not surpass, those achieved via traditional craniotomies and are associated with notably lower morbidity rates. This surgical approach, by significantly reducing the incidence of postoperative complications, not only enhances the patient recovery trajectory but also substantially alleviates the healthcare system’s burden associated with the management of such adverse events. The implications of these advancements are manifold, encompassing improved patient prognoses, more judicious use of medical resources, and a reduction in the length of hospital stays and associated healthcare costs.

In the evaluation of surgical planning, the integration of AR and VR technologies has emerged as a groundbreaking development. These technologies provide surgeons with an unprecedented level of detail regarding the spatial relationships between the tumor and critical neurovascular structures, enabling the precise tailoring of the surgical approach to the intricate anatomical configuration of each patient. Such precision is paramount in minimizing intraoperative risks and enhancing the likelihood of complete tumor resection while preserving neurological function. Preoperative planning with AR and VR can further enhance the benefits, indications, and applications of SOKCs. AR/VR shows potential benefits beyond preoperative planning. It has intraoperative implications and can minimize postoperative complications. Stronger evidence is needed for the use of AR/VR in clinical practice.

## Figures and Tables

**Table 1:** Detailed account of the surgical procedure.

Step	Procedure
1. Patient Positioning	The patient is positioned supinely.
	General anesthesia is administered.
	The head is elevated approximately 15 degrees and retro-flexed 25 to 30 degrees.
	A Mayfield head holder is used to stabilize the head.
2. Skin Incision	A precise incision is made without shaving, preserving cosmetic appearance and providing a controlled entry point (measuring around 2.5 to 3 cm in length).
3. Subcutaneous Dissection	Subcutaneous dissection: performed using monopolar electrocautery.
	Subperiosteal dissection: remove the galea from the squamous portion of the frontal bone
4. Craniotomy	A 2.5 cm × 2 cm “D-shaped” craniotomy is executed, facing the linear part downwards, along the anterior cranial fossa floor.
5. Drilling	The internal part of the orbital portion of the frontal bone is smoothed, optimizing the surgical field for tumor access.
6. Dural Opening	The dura is carefully opened in an oblique or cross fashion, exposing the underlying brain and meningioma.
7. Tumor Identification	Precise identification of the tumor borders and boundaries is performed to lay the groundwork for a successful resection.
8. Gentle Retraction	Rhoton forceps, cotton balls, and cotonoids are used, providing a clear line of sight to the tumor.
9. Arachnoid Layer Opening	To release cerebrospinal fluid (CSF) and alleviate surrounding pressure.
	Crucial for deeply seated lesions and widening the field.
10. Dissection with Neuronavigation	Meticulous dissection is aided by neuronavigation for precision and safety.
	Nearby neurovascular structures are gently retracted using microsurgery principles.
11. Hemostasis Review	Ensures meticulous control of bleeding sources, minimizing the risk of a postoperative hemorrhage.
12. Plane Closure	The surgical site is meticulously closed in layers to promote wound healing and reduce the risk of complications.

**Table 2:** Advantages and disadvantages of supraorbital keyhole craniotomy.

Aspect	Advantages	Disadvantages or Possible Complications
Patient positioning and preparation	Appropriate patient positioning fosters “retractorless” surgery	Time-consuming, and not readily available in community hospitals and low- and middle-income countries Anesthesia-related risks include allergic reactions, respiratory issues, or adverse reactions to medications
Skin incision, dural and arachnoid layer opening, and plane closure	Minimal cosmetic deformities	Cerebrospinal fluid (CSF) leak (4.3%) [23,24] Supraorbital hypesthesia (1.6% temporary, 4.3% permanent hypesthesia) [23,24] Facial nerve palsy (1% temporary, 2.9% permanent) [23,24] Surgical site infection (1%) [23,24] Cosmetic concerns including alopecia at the site of the incision (1%) [23,24] Allergic reactions to surgical materials, medications, or anesthesia
Surgical approach	Shorter hospital stays and faster recovery compared to traditional methods Precise access and minimal brain retraction reduces trauma to adjacent healthy tissue Drilling the inner edge of the craniotomy provides an additional working angle for deep-seated lesions	Damage to nearby neurovascular structures, resulting in potential stroke or other vascular complications. Limited view through the microscope; however, this can be helped by new microscopes with a flush opening, an endoscope, or performing an orbital roof osteotomy increasing space and exposure
Postoperative care	Preservation or improvement of neurological function, depending on the tumor’s location	Recurrence of the tumor due to subtotal resection Neurological deficits Postoperative pain, discomfort, and the need for pain management during recovery. Potential need for additional treatments such as radiation therapy, especially in cases of subtotal tumor resection Blood clots or deep vein thrombosis (DVT) due to reduced mobility during the recovery period Need for Intensive Care Unit (ICU) intubation Death

## Data Availability

The original contributions presented in this study are included in the article; further inquiries can be directed to the corresponding author.

## Abbreviations

Three-dimensional (3D), four-dimensional (4D), virtual reality (VR), augmented reality (AR), and Mixed Reality (MR).
